# Impact of active screening for methicillin-resistant *Staphylococcus aureus* (MRSA) and decolonization on MRSA infections, mortality and medical cost: a quasi-experimental study in surgical intensive care unit

**DOI:** 10.1186/s13054-015-0876-y

**Published:** 2015-04-08

**Authors:** Yuarn-Jang Lee, Jen-Zon Chen, Hsiu-Chen Lin, Hsin-Yi Liu, Shyr-Yi Lin, Hsien-Ho Lin, Chi-Tai Fang, Po-Ren Hsueh

**Affiliations:** Division of Infectious Diseases, Department of Internal Medicine, Taipei Medical University Hospital, 252 Wusing Street, Taipei, 11031 Taiwan; Graduate Institute of Clinical Medicine, College of Medicine, Taipei Medical University, 250 Wusing Street, Taipei, 11031 Taiwan; Department of Infection Control, Taipei Medical University Hospital, 252 Wusing Street, Taipei, 11031 Taiwan; Department of Pediatrics, School of Medicine, College of Medicine, Taipei Medical University, 250 Wusing Street, Taipei, 11031 Taiwan; Department of Laboratory Medicine, Taipei Medical University Hospital, 252 Wusing Street, Taipei, 11031 Taiwan; Department of General Medicine, School of Medicine, College of Medicine, Taipei Medical University, 250 Wusing Street, Taipei, 11031 Taiwan; Institute of Epidemiology and Preventive Medicine, College of Public Health, National Taiwan University, 17 Xu-Zhou Road, Taipei, 10002 Taiwan; Department of Internal Medicine, National Taiwan University Hospital, 7 Chun-Shan South Road, Taipei, 10002 Taiwan; Department of Laboratory Medicine, National Taiwan University Hospital, National Taiwan University College of Medicine, 1 Jen-Ai Road, Taipei, 10055 Taiwan

## Abstract

**Introduction:**

Methicillin-resistant *Staphylococcus aureus* (MRSA) is a leading pathogen of healthcare-associated infections in intensive care units (ICUs). Prior studies have shown that decolonization of MRSA carriers is an effective method to reduce MRSA infections in ICU patients. However, there is currently a lack of data on its effect on mortality and medical cost.

**Methods:**

Using a quasi-experimental, interrupted time-series design with re-introduction of intervention, we evaluated the impact of active screening and decolonization on MRSA infections, mortality and medical costs in the surgical ICU of a university hospital in Taiwan. Regression models were used to adjust for effects of confounding variables.

**Results:**

MRSA infection rate decreased from 3.58 (baseline) to 0.42‰ (intervention period) (*P* <0.05), re-surged to 2.21‰ (interruption period) and decreased to 0.18‰ (re-introduction of intervention period) (*P* <0.05). Patients admitted to the surgical ICU during the intervention periods had a lower in-hospital mortality (13.5% (155 out of 1,147) versus 16.6% (203 out of 1,226), *P* = 0.038). After adjusting for effects of confounding variables, the active screening and decolonization program was independently associated with a decrease in in-hospital MRSA infections (adjusted odds ratio: 0.3; 95% CI: 0.1 to 0.8) and 90-day mortality (adjusted hazard ratio: 0.8; 95% CI: 0.7 to 0.99). Cost analysis showed that $22 medical costs can be saved for every $1 spent on the intervention.

**Conclusions:**

Active screening for MRSA and decolonization in ICU settings is associated with a decrease in MRSA infections, mortality and medical cost.

**Electronic supplementary material:**

The online version of this article (doi:10.1186/s13054-015-0876-y) contains supplementary material, which is available to authorized users.

## Introduction

Methicillin-resistant *Staphylococcus aureus* (MRSA), first reported from England in 1961, is a leading pathogen of nosocomial infections in intensive care units (ICUs) [1,2]. In recent years, reduced susceptibility to vancomycin has made MRSA more difficult to treat than before [3,4]. Patients who have healthcare-associated MRSA (HA-MRSA) infections have increased mortality risk and prolonged hospital stay, resulting in increased medical costs, compared with patients who do not have HA-MRSA infections [5].

A significant proportion of MRSA infections are endogenous and are caused by the same strain that colonizes the nasal mucosa [6,7]. Observational studies [8-12] and the REDUCE MRSA trial [13] have consistently shown that decolonization of ICU patients, using intra-nasal mupirocin and chlorhexidine body-washing, can reduce MRSA infection rates. Decolonization directly reduces endogenous infections in carriers, and indirectly reduces exogenous infections in non-carriers. Nevertheless, whether the ultimate goals of infection control, that is, the reduction of medical cost and mortality, can be achieved by these sorts of interventions remains unsettled, as previous studies did not look for these outcomes [14,15].

In Taiwan, MRSA was first reported in the 1980s [16]. The proportion of MRSA among all *S. aureus* isolates that cause infections in ICUs has increased to approximately 80% [16,17]. In our hospital, MRSA infection rates in the ICU remained high, despite efforts on contact isolation and decolonization of patients with clinical MRSA infections. To control the problem, a routine active MRSA screening and decolonization program was implemented in the surgical ICU (SICU), which led to a rapid drop in MRSA infection rate. The program was temporarily suspended between May 2008 and August 2009, owing to a lack of financial support, followed by a resurge in MRSA infection rate. The program was then restarted in September 2009, and the MRSA infection rate rapidly decreased again.

Using a quasi-experimental study design, we sought to evaluate the impact of active screening and decolonization of ICU patients, including both direct and indirect protective effect, on the incidence of MRSA infections, mortality and medical costs.

## Methods

### Setting

This study was conducted in the SICU of Taipei Medical University Hospital (TMUH), a tertiary care, university-affiliated teaching hospital in northern Taiwan. TMUH has a 702-bed capacity. The SICU has 18 beds (all are single bed rooms).

### Ethical statement

The institutional review board (IRB) of TMUH approved the study protocol (protocol number: TMUH-05-11-04). The IRB approved the waiver of informed consent (see Additional file 1).

### Study design

This was a quasi-experimental, interrupted time-series study [18]. Regression models were used to adjust for the effects of confounding variables, including hospital-level infection-control practices (hand hygiene and bundle care) and patient-level risk factors (invasive procedures and severity of underlying diseases). Data on MRSA infection rate, mortality and medical cost were retrospectively obtained from computer databases.

**Figure 1:**
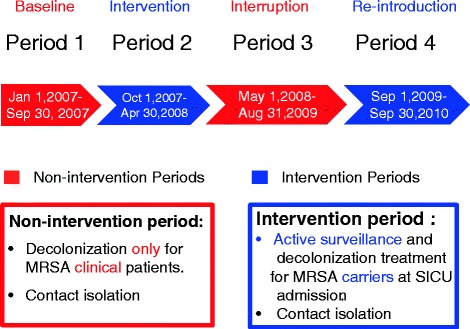
**Study design: period one (baseline), period two (intervention period), period three (interruption period) and period four (re-introduction of intervention period).** MRSA: methicillin-resistant *Staphylococcus aureus*; SICU: surgical intensive care unit.

The study period was divided into four stages. In period one (baseline, between January and September 2007), contact precautions, eradication and environmental disinfection at discharge were performed only for those patients with positive clinical cultures for MRSA. In period two (intervention period), routine active screening and decolonization (supported by a research grant from the hospital) was initiated and lasted between October 2007 and April 2008. The intervention was halted in period three (interruption period, between May 2008 and August 2009) owing to a lack of research grants. After a resurgence in the SICU MRSA infection rates during period three prompted the hospital leadership to provide financial support for the active screening and decolonization program, the intervention was resumed in period four (reintroduction period, between September 2009 and September 2010) (Figure 1).

We compared the HA-MRSA infection rates and mortality rates [5] of all patients admitted during the intervention periods (period two and period four) to those of patients admitted during the non-intervention periods (period one and period three), after adjusting for effects of other variables that may influence the outcomes.

### Study interventions

The intervention consisted of active surveillance cultures that were immediately taken from the anterior nares of the patients at the time of admission to the SICU to identify asymptomatic MRSA carriers. Extra-nasal cultures were not obtained. Before culture results became available, patients were put in a single-bed room and standard precautions were applied. If nasal swab cultures were positive, MRSA was eradicated from the nares by the application of mupirocin ointment (GlaxoSmithKline, GSK, Crawley, United Kingdom) three times per day for five days, and from the skin by the application of 4% chlorhexidine gluconate (Panion & BF Biotech Incorporation, Taoyuan, Taiwan) once per day for five days; contact precautions were also taken. Patients were screened for MRSA carriage only at their admission, rather than regularly during their stay in ICU.

### Microbiological procedures

During the intervention periods (period two and period four), the nasal swab was plated on blood agar plates, which were incubated at 35°C with 5% CO_2_ in ambient air, and were checked for the presence of *S. aureus* after 16 to 18 hours of incubation. To shorten the time interval from culture to reporting MRSA, suspected *S. aureus* isolates were tested for the presence of coagulase using BD BBL Coagulase Plasma Rabbit with EDTA (BD, Franklin Lakes, NJ, USA) (35°C overnight), and plated on oxacillin screen agar plates containing 6 μg/ml oxacillin and 4% NaCl (BD, Franklin Lakes, NJ, USA) at 35°C for 24 hours to test for oxacillin resistance.

### Hospital-level infection control practices

Hand hygiene practice continued to improve during the study period. The increase in hand washing was measured by the amounts of alcoholic disinfectant used for hand washing per 1,000 patient days (15.7 liters in period 1, 16.4 liters in period 2, 21.9 liters in period 3 and 23.4 liters in period 4), based on hospital administrative data.

There was no change in antibiotic prescribing patterns or overall sepsis management during the study period. No cohorting was used. Ventilator-associated pneumonia (VAP) bundle care, including head elevation, daily interruption of sedation for assessing extubation, and daily 0.2% chlorhexidine mouth cleansing, started in August 2009.

### Individual patient-level risk factors

Data on the severity of underlying diseases, including Acute Physiology and Chronic Health Evaluation II (APACHE II) score upon admission to the ICU, length of hospital stay before ICU admission and invasive procedures, were routinely recorded. We retrospectively obtained patient-level data from the hospital’s computer system database. The APACHE II scores were divided into two groups (low and high), using a cutoff point of 15 [19].

### Outcome ascertainment

In the study hospital, infection control nurses routinely review all hospitalizations for all types of healthcare-associated infections (HAIs) using the Centers for Disease Control and Protection (CDC) surveillance definitions [20,21]. The surveillance practice for detecting the HAIs remained the same throughout the study period. Data on HA-MRSA cases that occurred during the study period were retrospectively obtained from the routine surveillance records. The SICU HA-MRSA infection rate was defined as the number of HA-MRSA infections per 1,000 patient SICU days. In-hospital HA-MRSA infection rate was defined as the number of HA-MRSA infections per 1,000 patient days from the time of SICU admission to discharge.

The in-hospital mortality of the patients was ascertained using hospital medical records and death certificates. The 90-day mortality (from SICU admission, including deaths occurring after discharge) of the patients was ascertained using the National Death Registry [5], updated to the end of 2010. To protect the privacy of patients, personal identification numbers were scrambled and anonymized before database linkage.

### Medical cost

Data on length of hospital stay and medical costs were obtained from the National Health Insurance Claims database [5] of the study hospital. The list of medical costs included the costs for physician care, accommodations, nursing care, pharmacy services, laboratory procedures, operations, rehabilitation programs, medications and anesthesia.

### Cost of intervention

All costs were calculated using an exchange rate of US$1 = NT$30. The costs for active screening included the cost of the materials for microbiological procedures (US$5 (NT$150) per ICU patient) and the cost of nursing personnel (US$6.7 (NT$200)/hour × 5 minutes per ICU patient) and laboratory technicians (US$5 (NT$150)/hour × 10 minutes per patient). The cost for decolonization included the cost of mupirocin (1 tube at US$1.7 (NT$51), per MRSA carrier) and chlorhexidine (20 ml at US$0.19 (NT$5.8), per MRSA carrier).

The costs for contact isolation included the costs of gloves (US$0.05 (NT$1.6) per unit), surgical masks (US$0.04 (NT$1.28) per unit), aprons (cleansing and disinfection) (US$0.003 (NT$0.08) per unit) and alcohol disinfectants for hand washing (10 ml at US$0.12 (NT$3.64), after each contact). There were, on average, fifty contacts per day (including forty nursing contacts, two physician contacts, four respiratory therapist contacts, two nursing specialist contacts, and two paramedic contacts), for a total of fourteen days of isolation for each MRSA carrier. We assumed a 100% adherence to contact precautions, with gloves, mask and apron changed after each contact, as well as hand washing.

### Statistical analyses

All analyses were conducted using SAS version 9.2 (SAS Institute, Cary, NC, USA). Risk factors for MRSA infections or mortality were analyzed using logistic regression or Cox regression. For multivariate analyses, all potential risk factors were included in the maximum model. We used stepwise regression procedures to identify independent risk factors. All tests were two-tailed, and *P* <0.05 was considered to be statistically significant.

### Number of MRSA infections averted

We estimated the number of MRSA infections that would have occurred in the absence of the intervention program using the following formula:

Total patient days in period two × (observed MRSA infection rate in period two/adjusted hazard ratio of intervention) + Total patient days in period four × (observed MRSA infection rate in period four/adjusted hazard ratio of intervention).

The number of MRSA infection cases averted by the intervention program was estimated using the predicted numbers (in the absence of the program) in Periods 2 and 4 minus the observed number in Periods 2 and 4.

### Cost-saving analysis

The cost saved by the intervention was estimated by multiplying the number of averted MRSA infections with the median excess total hospitalization for each MRSA infection case. We estimated the cost saved for every dollar spent on active screening and decolonization program by dividing the cost saved for the averted MRSA infections by the cost of implementing the intervention.

## Results

### Baseline characteristics

A total of 2,373 patients were admitted to the SICU during the study period. Table 1 shows the baseline characteristics of the patients admitted during the different periods.

**Table 1:** **Baseline characteristics of the 2,373 patients admitted to the surgical ICU**

**Variable**	**Period 1 (non-intervention) (n = 327)**	**Period 2 (intervention) (n = 314)**	***P*** **value**	**Period 3 (non-intervention) (n = 899)**	**Period 4 (intervention) (n = 833)**	***P*** **value**
Age (mean ± SD)	62 ± 18	65 ± 17	0.060	61 ± 18	62 ± 18	0.234
Age >65 (years)	163 (49.8)	177 (56.4)	0.098	424 (52.9)	408 (51.1)	0.450
Male	204 (62.4)	189 (60.1)	0.569	543 (60.4)	481 (57.7)	0.261
Pre-ICU LOS (mean ± SD)	4.1 ± 7.7	5.5 ± 12.4	0.104	3.5 ± 7.4	3.2 ± 6.7	0.310
APACHE II (median)	16	15	0.368	10	10	0.263
APACHE II >15	182 (55.7)	157 (50)	0.151	271 (30.2)	260 (31.3)	0.630
Endotracheal intubation	58 (17.7)	50 (15.9)	0.540	124 (13.7)	102 (12.2)	0.340
Operation	210 (64.2)	229 (72.9)	0.018	680 (75.6)	594 (71.3)	0.036
CVC catheter	164 (50.1)	169 (53.8)	0.353	451 (50.1)	395 (47.4)	0.253
Foley catheter	128 (39.1)	104 (33.1)	0.113	300 (33.3)	305 (36.6)	0.157
Double lumen catheter	11 (3.4)	11 (3.5)	0.923	36 (4)	27 (3.2)	0.397

Data are number (%) unless otherwise specified. APACHE II: Acute Physiology and Chronic Health Evaluation II; CVC: Central venous catheter; Pre-ICU LOS: before ICU length of hospital stay (days).

### Active surveillance cultures

Of the 314 patients admitted in period 2, 213 (67.8%) received active screening for MRSA. The remaining 101 patients did not receive such surveillance cultures because of mortality soon after admission or rapid transfer to other wards. Similarly, of the 833 patients admitted in period 4, 538 (64.5%) received surveillance cultures. The average ICU-admission-to-culture-reporting time was 2.5 days. Among surveillance cultures, the positive rate in periods 2 and 4 was 11.3% (24 out of 213) and 6.1% (33 out of 538), respectively (*P* = 0.028).

### Healthcare-associated MRSA infection rates in the surgical ICU

**Figure 2:**
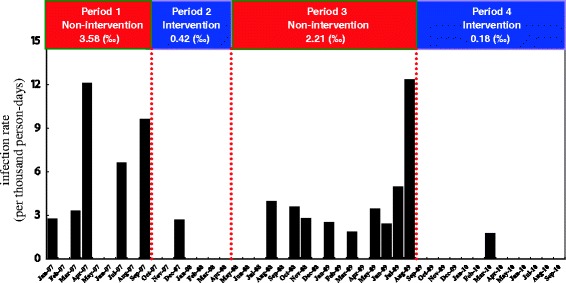
**Monthly incidence of healthcare-associated methicillin-resistant** ***Staphylococcus aureus*** **infections during non-intervention periods (period one and period three) and intervention periods (period two and period four) in the surgical intensive care unit.**

Twenty-three patients developed MRSA infections (including eleven bloodstream infections, eight respiratory tract infections, two urinary tract infections, one cardiovascular system infection and one eye, ear, nose, throat or mouth infection) during their stay in the SICU in non-intervention periods, compared with two patients (two respiratory tract infections) during the intervention periods (Table 2). After the start of intervention, the monthly MRSA infection rate in the SICU rapidly dropped to zero (Figure 2) (overall MRSA infection rate: 3.58‰ (period 1) versus 0.42‰ (period 2), *P* <0.05). After the suspension of the program in May 2008, the monthly MRSA infection rates in the SICU rapidly resurged and rose to 12‰ in August 2009 (Figure 2), despite an improved hand hygiene practice from 16.44 to 21.87 liters per 1,000 patient days. After re-introduction of the intervention program in September 2009, the monthly MRSA infection rate rapidly dropped to zero again (Figure 2) (overall MRSA infection rate: 2.21‰ (period 3) versus 0.18‰ (period 4), *P* <0.05).

**Table 2:** **Healthcare-associated MRSA infection rates**

**Variable**	**Period 1 Non-intervention (n = 327)**	**Period 2 Intervention (n = 314)**	**Period 3 Non-intervention (n = 899)**	**Period 4 Intervention (n = 833)**
Total patient days (SICU)	2,792	2,371	5,875	5,434
Total patient days (in-hospital)	7,028	6,817	17,387	16,523
% of patients screened at ICU admission	-	67.8% (213/314)	-	64.5% (538/833)
MRSA positive rate of surveillance culture	-	11.3% (24/213)	-	6.1% (33/538)
MRSA infection rate (‰) (SICU)	3.58	0.42	2.21	0.18
SICU MRSA infection (number)	10	1^a^	13	1^a^
BSI infection	6	0	5	0
RTI infection	3	1	5	1
UTI infection	0	0	2	0
SSI infection	0	0	0	0
Other infection sites (CVSI, EENTI)	1	0	1	0
MRSA infection rate (‰) (in-hospital)	1.42	0.29	0.75	0.24
In-hospital MRSA infection (number)	10	2^b^	13	4^b^

^a^Both cases had negative surveillance culture at the time of SICU admission. ^b^Including the two SICU MRSA infection cases. Of the four MRSA infection cases that occurred after transfer to general wards, three cases had negative surveillance culture at the time of SICU admission, and the other had positive surveillance culture at the time of SICU admission. BSI: Bloodstream infection; CVSI: Cardiovascular system infection; EENTI: eye, ear, nose, throat, or mouth infection; MRSA: Methicillin-resistant *Staphylococcus aureus*; RTI: Respiratory tract infection; SICU: Surgical intensive care unit; SSI: Surgical site infection; UTI: Urinary tract infection.

### Effect of intervention on in-hospital MRSA infections

Univariate logistic regression analysis showed that the active screening and decolonization program was a significant protective factor for in-hospital MRSA infections (Table 3). Multiple logistic regression analysis showed that the intervention was an independent protective factor (adjusted odds ratio (OR): 0.3; 95% CI: 0.1 to 0.8) (Table 3).

**Table 3:** **Risk factors for in-hospital MRSA infections**

**Univariate logistic regression analysis**			**Multiple logistic regression analysis**	
**Variable**	**OR (95% CI)**	***P*** **value**	**Adjusted OR (95% CI)**	***P*** **value**
Age >65 (years)	2.1 (0.9-4.7)	0.077		
Sex	1.0 (0.5-2.1)	0.961		
Pre-ICU LOS	1.0 (1.0-1.1)	0.148		
Operation	1.4 (0.5-3.3)	0.513		
CVC catheter	1.7 (0.8-3.8)	0.166		
Foley catheter	3.2 (1.5-7.0)	0.004		
Double lumen catheter	3.5 (1.0-12.0)	0.042		
APACHE II >15	3.0 (1.4-6.5)	0.007	2.6 (1.1-6.0)	0.025
Endotracheal intubation	4.3 (2.0-9.4)	0.0001	3.5 (1.6-8.0)	0.002
Antibiotic use	1.4 (0.6-3.1)	0.469		
Hand hygiene^a,b^	0.9 (0.8-1.0)	0.029		
VAP bundle	0.3 (0.1-0.8)	0.021		
Intervention	0.3 (0.1-0.8)	0.009	0.3 (0.1-0.8)	0.015

^a^Hand hygiene: the amount of alcoholic disinfectant (liter) used for hand washing per 1,000 patient days during a period. ^b^ *P* = 0.569 for the interaction between intervention and hand hygiene. APACHE II: Acute Physiology and Chronic Health Evaluation II; CVC: central venous catheter; ICU LOS: ICU length of hospital stay; OR: odds ratio; MRSA: methicillin-resistant *Staphylococcus aureus*; VAP: ventilator-associated pneumonia.

Univariate Cox regression analysis showed that the active screening and decolonization program was a significant protective factor for MRSA infections (Table 4). Multiple Cox regression analysis showed that the intervention was an independent protective factor (adjusted hazard ratio (HR): 0.3; 95% CI: 0.1 to 0.7) (Table 4).

**Table 4:** **Risk factors for time to in-hospital MRSA infections**

**Univariate Cox regression analysis**			**Multiple Cox regression analysis**	
**Variable**	**HR (95% CI)**	***P*** **value**	**Adjusted HR (95% CI)**	***P*** **value**
Age >65 (years)	1.5 (0.7-3.4)	0.307		
Sex	1.0 (0.5-2.2)	0.972		
pre-ICU LOS	1.0 (1.0-1.0)	0.680		
Operation	1.1 (0.4-2.6)	0.913		
CVC catheter	1.1 (0.5-2.4)	0.858		
Foley catheter	1.0 (0.4-2.3)	0.994		
Double lumen catheter	1.5 (0.4-5.2)	0.505		
APACHE II >15	2.1 (1.0-4.6)	0.066	2.3 (1.0-5.3)	0.037
Endotracheal intubation	1.7 (0.8-3.9)	0.177		
Antibiotic use	1.2 (0.5-2.8)	0.657		
Hand hygiene^a,b^	0.9 (0.8-1.0)	0.051		
VAP bundle	0.3 (0.1-0.9)	0.025		
Intervention	0.3 (0.1-0.7)	0.010	0.3 (0.1-0.7)	0.006

^a^Hand hygiene: the amount of alcoholic disinfectant (liter) used for hand washing per 1,000 patient days during a period. ^b^ *P* = 0.597 for the interaction between intervention and hand hygiene. APACHE II: Acute Physiology and Chronic Health Evaluation II; CVC: central venous catheter; HR: hazard ratio; ICU LOS: ICU length of hospital stay; MRSA: methicillin-resistant *Staphylococcus aureus*; VAP: ventilator-associated pneumonia.

### In-hospital mortality

The in-hospital mortality rate of patients admitted to the SICU was 19.3% (63 out of 327) in period 1, which decreased to 13.7% (43 out of 314) in period 2 (intervention period), increased again to 15.6% (140 out of 899) in period 3 (interruption period) and decreased again to 13.4% (112 out of 833) in period 4 (re-introduction period). Patients admitted to the SICU during the intervention periods had a significantly lower in-hospital mortality than those admitted during the non-intervention periods (13.5% (155 out of 1,147) versus 16.6% (203 out of 1,226); *P* = 0.038; chi-square test).

### Effect of intervention on 90-day mortality

Table 5 shows the results of Cox regression analysis for risk factors of mortality within 90 days. Multiple Cox regression analysis showed that the intervention was an independent protective factor against mortality (adjusted HR: 0.8; 95% CI: 0.7 to 0.99), after adjusting for the effects of the other variables (Table 5).

**Table 5:** **Risk factors for 90-day mortality**

**Univariate Cox regression analysis**			**Multiple Cox regression analysis**	
**Variable**	**HR (95% CI)**	***P*** **value**	**Adjusted HR (95% CI)**	***P*** **value**
Age >65 (years)	2.4 (1.9-2.8)	<0.0001	1.5 (1.2-1.8)	0.0002
Sex	1.0 (0.8-1.1)	0.730		
pre-ICU LOS	1.0 (1.0-1.0)	<0.0001	1.0 (0.99-1.0)	0.421
Operation	0.4 (0.3-0.5)	<0.0001	0.3 (0.3-0.4)	<0.0001
CVC catheter	1.6 (1.3-1.9)	<0.0001	1.5 (1.2-1.9)	0.0002
Foley catheter	2.6 (2.2-3.2)	<0.0001	1.4 (1.2-1.7)	0.0007
Double lumen catheter	4.3 (3.2-5.7)	<0.0001	1.7 (1.2-2.3)	0.001
APACHE II >15	3.8 (3.1-4.5)	<0.0001	2.5 (2.0-3.0)	<0.0001
Endotracheal intubation	3.8 (3.1-4.5)	<0.0001	1.5 (1.2-1.9)	0.0003
Hand hygiene^a,b^	0.95 (0.9-0.98)	0.002	1.0 (0.9-1.0)	0.617
Intervention	0.9 (0.7-1.1)	0.219	0.8 (0.7-0.99)	0.048

^a^Hand hygiene: the amount of alcoholic disinfectant (liter) used for hand washing per 1,000 patient days during a period. ^b^ *P* = 0.16 for the interaction between intervention and hand hygiene. APACHE II: Acute Physiology and Chronic Health Evaluation II; CVC: central venous catheter; HR: hazard ratio; ICU LOS: ICU length of hospital stay.

### Excess length of hospital stay

Mean length of hospital stay was significantly higher for patients with MRSA infections than for those without MRSA infections (in SICU: 40.6 days versus 6.6 days; total hospitalization: 75.4 days versus 23.3 days; both *P* <0.001), with an excess of 34.0 days (in SICU) and 52.1 days (total hospitalization) (Table 6).

**Table 6:** **Comparison of hospital stay and hospital cost for patients with or without MRSA infections**

**Category**	**Cases with MRSA infections**	**Cases without MRSA infections**	***P*** **value**
Length of stay (days)			
SICU length of stay (mean)	40.6	6.6	<0.001
SICU length of stay (median)	37	2	<0.001
Total length of hospital stay (mean)	75.4	23.3	<0.001
Total length of hospital stay (median)	59	15	<0.001
Cost^a,b^			
SICU costs (mean)	25,466	6,612	<0.001
SICU costs (median)	25,161	3,748	<0.001
Total hospitalization costs (mean)	31,815	8,505	<0.001
Total hospitalization costs (median)	31,020	4,961	<0.001

^a^in US dollars (exchange ratio US$1 = NT$30). ^b^The medical cost, including physician care, accommodation, nursing care, meals, laboratory procedures, treatments, operations, rehabilitation programs, medications, pharmacy service and anesthesia. MRSA: methicillin-resistant *Staphylococcus aureus*; SICU: surgical intensive care unit.

### Excess cost for patients with MRSA infections

The mean hospital cost was significantly higher for patients with MRSA infections than for those without MRSA infections (in SICU: US$25,466 versus US$6,612; total hospitalization: US$31,815 versus US$8,505; both *P* <0.001), with an excess of US$18,854 (in SICU) and US$23,310 (total hospitalization) (Table 6).

### Cost-saving by intervention

The number of MRSA cases prevented by the intervention was estimated to be 13 (6,817 patient days (period 2) × 0.00029 (observed MRSA infection rate in period 2)/0.3 (adjusted HR of intervention) + 16,523 patient days (period 4) × 0.00024 (observed MRSA infection rate in period 4)/0.3 (adjusted HR of intervention)).

The cost saved by preventing 13 MRSA cases during the total 20-month intervention period (periods 2 and 4) was 13 × US$23,310 = US$303,030 (annual cost saving: US$190,908). The active surveillance and decolonization program cost US$13,717 during the total 20-month intervention period (annual cost: US$8,231). The cost-saving ratio was 22 (US$303,030/US$13,717); thus, every dollar spent on the intervention resulted in a saving of $22 in medical costs.

## Discussion

Our results show that the active screening and decolonization program was associated with a decrease in all-type clinical in-hospital MRSA infections (adjusted OR: 0.3; 95% CI: 0.1 to 0.8) and a lower 90-day mortality (adjusted HR: 0.8; 95% CI: 0.7 to 0.99). Furthermore, the active screening and decolonization program is cost-saving; every dollar spent on interventions resulted in a saving of $22 in medical costs.

Due to ethical considerations, we were unable to use randomized controlled experiments to evaluate the active screening and decolonization program. Nevertheless, the interrupted time-series study design, in conjunction with the use of regression models to control the effects of hospital-level and patient-level confounders, strengthens the causal inference. The rapid drop, resurgence and drop again in MRSA infection rates, following introduction, interruption and re-introduction of the interventions in temporal sequence, makes a strong case against alternative explanations such as a progressive decline in MRSA carrier rates in community, or a continuing improvement in overall hospital infection control measures. Furthermore, after adjusting for effects of hospital-level improvements in infection-control practices (hand hygiene and bundle care), as well as individual patient-level risk factors (invasive procedures and severity of underlying diseases), the intervention remains an independent protective factor against MRSA infection and mortality.

The protective effect measured in our study includes both the direct effect and the indirect effect. Decolonization of MRSA carriers directly reduces his or her risk of subsequent MRSA infections. Moreover, decolonization of MRSA carriers prevents the transmission of MRSA to non-carriers that would otherwise happen, and therefore indirectly protects those who are not carriers at the time of ICU admission.

The impact of active screening and decolonization on MRSA transmission within hospitals is further highlighted by a decrease in the prevalence of MRSA carriers among hospitalized patients. Mathematical modeling studies predict that, in settings where MRSA carriage is endemic, the implementation of active screening and decolonization will lead to a rapid drop in MRSA carriage rate in hospitalized patients [22]. As predicted by modeling studies, the average MRSA-positive rate among surveillance cultures in our study rapidly dropped from the initial 11.3% (24 out of 213; period 2) to 6.1% (33 out of 538; period 4) (*P* = 0.038). The 47% decrease in MRSA carriage rate (from 11.3 to 6.1%) within a short time period is consistent with the effect of the active screening and decolonization program in period 2 on blocking nosocomial MRSA transmission [22].

The use of intranasal mupirocin and chlorhexidine baths for decolonization in our study is likely the key factor of the observed efficacy. Two recent cluster-randomized trials conclusively showed that active surveillance and isolation alone, in the absence of a decolonization program, did not reduce the MRSA-positive clinical culture rate or the MRSA bloodstream infection rate [13,23,24]. Although another study reported that universal surveillance, contact precaution and hand hygiene without decolonization were associated with a 62% decrease in MRSA infections in ICUs [25], an independent analysis of the data using mathematical modeling showed that only a very small fraction of the observed effect could be attributed to active detection and isolation alone [26].

MRSA infections increase the mortality of hospitalized patients by 12.4 to 28.5% [5]. Theoretically, prevention of HA-MRSA infections in ICU patients should lead to a reduction in mortality. However, the survival of ICU patients is heavily influenced by their acute severity of illnesses, prior length of stay and underlying diseases [19,27], which need to be taken into account in analyzing the effect of interventions on the mortality of ICU patients. We showed that, after adjusting for effects of the above-stated variables, the active screening and decolonization program is an independent protective factor against mortality. Thus, decolonization of MRSA is not only an infection-control measure, but also could be a potentially life-saving intervention for all ICU patients in settings with a high MRSA infection rate.

In the present study, we used inexpensive conventional screening plates to detect MRSA carriers, with a turnaround time of two to three days. The efficacy of interventions may be better if rapid tests were used for screening to minimize the delay in initiating decolonization. Tests based on real-time PCR have a turnaround time of less than one day, but are expensive [28]. Culture-based methods using chromogenic screening media can yield a rapid result after 18 to 24 hours incubation, but sensitivity varies by product, and is usually lower than that of PCR-based methods [29].

Because of logistic consideration, we did not obtain extra-nasal cultures when screening individuals for MRSA colonization. Use of nasal culture to guide decolonization in our study, however, appears to be sufficient to yield a significant decrease in MRSA infection rates and decrease in mortality. The additional use of extra-nasal cultures will likely detect more MRSA carriers, and thus increase the impact of the intervention [30].

Cost is an important concern for the sustained implementation of HAI prevention efforts. Consistent with previous cost analyses of MRSA prevention programs [31-33], our data show that, because of the high excess cost associated with MRSA infection and related complications (US$23,310 per MRSA infection case) in the SICU setting, the investment in active screening and decolonization (US$8,231 per year) will actually be cost-saving if at least one case of MRSA infection is prevented in the SICU every year. We estimated that 13 cases of MRSA infections had been prevented during the total 20-month intervention period, which yielded a highly beneficial cost-saving ratio of 22.

An alternative to the screening followed by targeted decolonization approach is universal decolonization for all ICU patients [12,34]. A multicenter cluster-randomized trial showed that daily chlorhexidine baths for all ICU patients reduced hospital-acquired bloodstream infection rates by 28% [34]. The REDUCE MRSA trial further demonstrated that universal administration of intranasal mupirocin and daily chlorhexidine baths in ICUs was more effective at reducing the MRSA clinical culture rate and bloodstream infection rate from any pathogen than was targeted decolonization [12]. Universal decolonization eliminates the problem of false negative screening results. There will be no delay in initiating decolonization [12]. Additional advantages include reduction in contact precaution, as well as reduction in infections caused by bacteria other than MRSA [12]. It is reasonable to expect that universal decolonization will have a greater effect in reducing mortality of ICU patients than targeted decolonization. However, an important concern for the universal use of mupirocin for all ICU patients is selection for mupirocin-resistant strains [24,35]. Moreover, for those ICU patients who do not carry MRSA, the use of mupirocin is arguably not justified. Until these concerns can be adequately addressed, targeted decolonization remains an important option for reducing MRSA-related morbidity and mortality in routine clinical settings.

Our study has several limitations. First, our results in the SICU may not be generalizable to medical ICUs, where patients generally have prolonged hospital stays and more complicated illnesses than patients in SICUs. Second, we did not conduct a molecular analysis of the MRSA strains isolated during the study periods to differentiate MRSA of endogenous origin from MRSA that was exogenously acquired through intra-hospital transmission. Additional limitations included the lack of follow-up cultures during the study period; thus, the effect of the mupirocin eradication of the MRSA in the carriers was not documented. Further monitoring of MRSA susceptibility to mupirocin is necessary to ensure the long-term efficacy of the program.

## Conclusions

Active screening for MRSA and decolonization in ICU settings with a high MRSA infection rate is associated with a decrease in MRSA infections, mortality and medical cost.

## Key messages

- Methicillin-resistant *Staphylococcus aureus* (MRSA) is a leading pathogen of healthcare-associated infections in intensive care units (ICUs).
- Routine active screening for MRSA and decolonization in ICU settings is associated with a decrease in MRSA infections, mortality and medical cost in settings with a high MRSA infection rate.
- Both MRSA carriers and non-carriers can benefit from a routine active MRSA screening and decolonization program.
- In settings where MRSA is endemic, MRSA carriage rate drops after implementation of the active screening and decolonization program.

## Additional file

Additional file 1:
**IRB certificate for approval.**

## Abbreviations

APACHE II: Acute Physiology and Chronic Health Evaluation II
HA: Hospital-acquired
HAI: Healthcare-associated infection
HA-MRSA: Healthcare-associated methicillin-resistant *Staphylococcus aureus*
HR: Hazard ratio
ICU: Intensive care unit
IRB: Institutional review board
MRSA: Methicillin-resistant *Staphylococcus aureus*
OR: Odds ratio
SICU: Surgical intensive care unit
TMUH: Taipei Medical University Hospital
VAP: Ventilator-associated pneumonia
