# Supplementary material for: Impact of active screening for methicillin-resistant Staphylococcus aureus (MRSA) and decolonization on MRSA infections, mortality and medical cost: a quasi-experimental study in surgical intensive care unit
Source: Crit Care. 2015 Apr 8;19(1):143. doi: 10.1186/s13054-015-0876-y (PMC4403941; doi:10.1186/s13054-015-0876-y)
Supplement: Additional file 1: — IRB certificate for approval. [file 13054_2015_876_MOESM1_ESM.pdf]

臺北醫學大學附設醫院 臨床(人體)試驗委員會
INSTITUTIONAL REVIEW BOARD OF TAIPEI MEDICAL UNIVERSITY HOSPITAL

110 台北市信義區吳興街 252 號
252, Wu Hsing Street, Taipei, Taiwan, R.O.C.
TEL:886-2-27372181-3749
FAX:886-2-55589908
E-mail : irb@h.tmu.edu.tw
日期：2011 年 5 月 27 日

研究計畫執行許可書

計畫名稱：	主動篩檢及介入措施對成人加護病房抗藥性金黃色葡萄球菌感染發生率之影響--回溯性世代研究。
計畫編號/本會編號：	TMUH-05-11-04
計畫主持人：	李垣樟
計畫起迄期間：	2011.05.01~2012.12.31
受試者知情同意書版本：	免受試者知情同意
許可書有效期間：	2011.05.27~2012.12.31

※ 依照 ICH-GCP 規定，臨床試驗每屆滿一年，人體試驗委員會必須定期重新審查臨床試驗後，方可繼續進行。
請於許可書有效期限到期二個月前繳交期中報告，以利本會進行審查。

Certificate of Approval

The following documents have been reviewed and approved by Institutional Review Board.

Protocol Title:	Effect of Active Surveillance and Intervention on Incidence of Methicillin-Resistant Staphylococcus aureus (MRSA) Infections in Adult Intensive Care Units--Retrospective Cohort Study.
Protocol No./ IRB No.:	TMUH-05-11-04
Principal Investigator:	Yuarn-Jang-Lee
Protocol Period:	2011.05.01~2012.12.31
Informed Consent Form:	Waiver
Valid Period of Certificate:	2011.05.27~2012.12.31

※ According to ICH-GCP, IRB will have to review each approved clinical research annually and decide whether it should be continued or not. Therefore, please send us (IRB) your Mid-term Report two months before the expiry date.

Sincerely yours,

Shyr-Yi Lin M.D. Ph.D.
Chairman
Institutional Review Board
Taipei Medical University Hospital
Taiwan, R.O.C.

Institutional Review Board
Taipei Medical University Hospital
Date 2011 / 5 / 27
